# Novel crab predator causes marine ecosystem regime shift

**DOI:** 10.1038/s41598-018-23282-w

**Published:** 2018-04-12

**Authors:** J. Kotta, T. Wernberg, H. Jänes, I. Kotta, K. Nurkse, M. Pärnoja, H. Orav-Kotta

**Affiliations:** 1 Estonian Marine Institute, University of Tartu, Mäealuse 14, 12618 Tallinn, Estonia; 2 UWA Oceans Institute & School of Biological Sciences, University of Western Australia, Perth, WA6009 Australia; 3 School of Life and Environmental Sciences, Centre for Integrative Ecology, Deakin University, Melbourne, VIC3125 Australia

## Abstract

The escalating spread of invasive species increases the risk of disrupting the pathways of energy flow through native ecosystems, modify the relative importance of resource (‘bottom-up’) and consumer (‘top-down’) control in food webs and thereby govern biomass production at different trophic levels. The current lack of understanding of interaction cascades triggered by non-indigenous species underscores the need for more basic exploratory research to assess the degree to which novel species regulate bottom-up and/or top down control. Novel predators are expected to produce the strongest effects by decimating consumers, and leading to the blooms of primary producers. Here we show how the arrival of the invasive crab *Rhithropanopeus harrisii* into the Baltic Sea – a bottom-up controlled ecosystem where no equivalent predators ever existed – appeared to trigger not only strong top-down control resulting in a decline in richness and biomass of benthic invertebrates, but also an increase in pelagic nutrients and phytoplankton biomass. Thus, the addition of a novel interaction – crab predation – to an ecosystem has a potential to reduce the relative importance of bottom-up regulation, relax benthic-pelagic coupling and reallocate large amounts of nutrients from benthic to pelagic processes, resulting in a regime shift to a degraded ecosystem state.

## Introduction

Ecosystems are characterised by the pathways of energy moving through food webs from producers to consumers. If these energy flows are disrupted, ecosystems can destabilize and transition into new configurations (or states) that have impoverished biological diversity and diminished ecological functions^1,2^. The socio-economic consequences of such regime shifts are profound when human and ecological health is compromised^1,3^.

The escalating spread of invasive species to new ecosystems increases the risk of introducing novel ecological functions that can shift the boundaries for biomass production at different trophic levels by disrupting the balance between resource and consumer control of native food webs^4–6^. The underlying mechanisms driving ecosystem structure are the opposing forces of bottom-up versus top down control, where resource availability (e.g. light and nutrients) sets theoretical limits for subsequent trophic levels by determining the consumable amount of biomass^7–9^ and consumption regulates the realized biomass at each trophic level^10,11^.

Decapod crabs are keystone species in many coastal food webs because they are able to move between spatially localized resources to exert control over benthic communities at relatively large spatial scales^12–14^. Most crabs are generalists with broad dietary preferences, although bivalves often make up the bulk of their diet^15^. As bivalves mediate substantial energy fluxes between benthic and pelagic habitats^16,17^, a reduction of bivalve populations by crabs could lead to new trophic pathways dominated by pelagic energy flows and the proliferation of pelagic primary producers. Although crabs are abundant in coastal habitats worldwide, their pervasiveness impedes the detection of their important ecological function at the ecosystem level.

The mud crab *Rhithropanopeus harrisii* (Gould, 1841) has its native distribution range from New Brunswick (Canada) to Veracruz (Mexico). The species was first found in Europe in 1874 in the Netherlands. Despite occasional observations in the Baltic Sea area as early as 1936, it was only in the late 1990’s and early 2000’s that the crab suddenly expanded into all basins of the Baltic Sea, although some sites still remain uncolonised^18^. The species was first discovered in Estonian waters in 2011 in Pärnu Bay, an area of regular prior surveys^19^. Before its introduction, there were no crabs or equivalent predators in most of the Baltic Sea and the majority of the coastal ecosystem was largely bottom-up regulated with the availability of nutrients, food and space controlling species abundances^20^.

The invasive mud crab is tolerant of a broad range of environmental conditions including the low salinity of the Baltic Sea. In its invasive range mud crabs occur from exposed hard bottoms to sheltered soft bottoms that are either unvegetated or vegetated^18^. Within these habitats the mud crab has a very broad diet potentially including macroalgae and benthic invertebrates^21^, either sessile and mobile species^22^, and the diet composition is largely driven by prey availability^23^. Currently, the Baltic Sea provides *R*. *harrisii* with a favourable habitat − a rich feeding ground with prey lacking defenses against predators as compared with environments where invertebrate prey have co-evolved with predatory crabs^24^. Moreover, the mud crab almost lacks natural enemies in its invaded range because commercially valuable predatory fishes are now depressed due to high fishing pressure^25^. Water birds might consume some mud crabs but due to high turbidity and low number of birds this control is not important in the study area^16^. This has resulted in a rapid dispersal and expansion of the mud crab populations after its initial invasion^18,19^.

In this study, we use a unique combination of long-term monitoring data of benthic and pelagic environments, targeted mud crab censuses, and manipulative field experiments to show that profound ecosystem changes are being caused by the recent introduction of the mud crab *R*. *harrisii* into the Baltic Sea, a bottom-up driven ecosystem where no equivalent predators ever existed. In doing so we demonstrate how the addition of a novel predator can trigger a regime shift through strong top-down control of suspension feeding invertebrates, modulating pelagic nutrient availability and increasing the magnitude and frequency of phytoplankton blooms.

## Results

Only one year after their initial discovery, mud crabs were found at densities between 1–3.2 individuals per artificial collector throughout Pärnu Bay. Within two years, the crab populations had expanded on average to more than 8 individuals per collector (with a local maximum of 43 individuals per collector) and extended their distribution more than 40 kilometers from the initial site of discovery (see Supplementary Appendix 1 online). The average carapace width of mud crabs was 1.2 cm.

**Figure 1:**
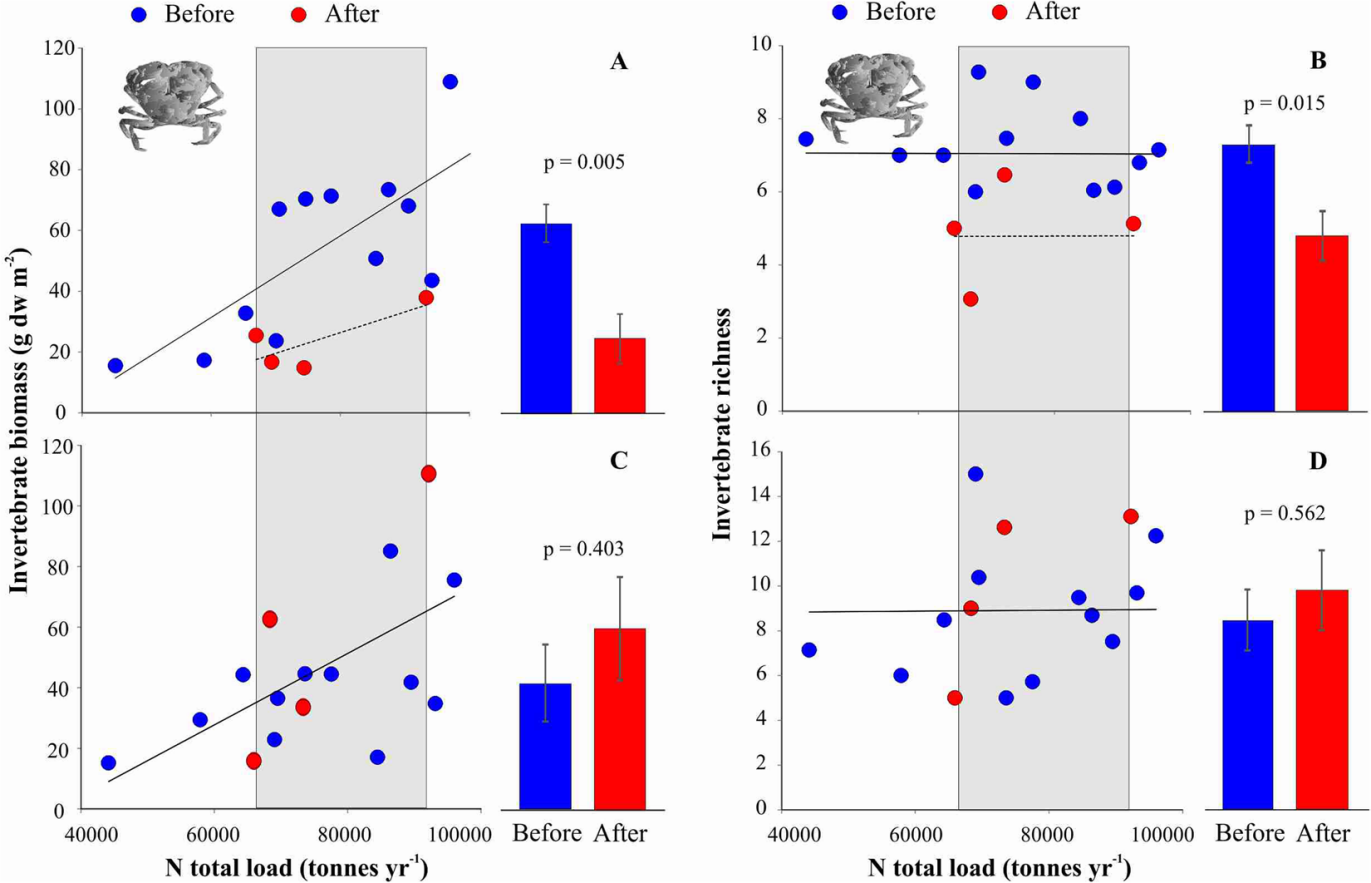
Relationship between annual nitrogen load, total biomass and richness of benthic invertebrates on soft bottom habitat before and after crab invasion in crab-infested (**A**,**B**) and control areas (**C**,**D**). Fitted lines highlight the slope of the linear regressions. Grey area shows the nutrient load values after crab invasion and this range was used to compare changes in the biota. Vertical bars denote 95% confidence intervals.

Prior to the arrival of the crab there was a positive relationship between annual nutrient load and total biomass, but not richness, of benthic invertebrates in Pärnu Bay. However, after the invasion of the mud crab, the relationship between nutrient loading and benthic invertebrate biomass became notably weaker (Fig. 1) and benthic invertebrate biomass (Fig. 1A) and invertebrate richness (Fig. 1B) declined 61% and 35%, respectively. These effects were mostly attributed to a decline in biomass of the dominant native bivalve *Limecola balthica* (L.), and the disappearance of clams, cockles and gastropods, at the crab infested sites. In the same area, the biomass of the invasive bivalve *Dreissena polymorpha* (Pallas, 1771) doubled and the invasive polychaete *Laonome* sp. also appeared in high densities (see Supplementary Appendix 2 online). In control areas, not yet colonized by the crab, no such changes were detected (Fig. 1C,D).

**Figure 2:**
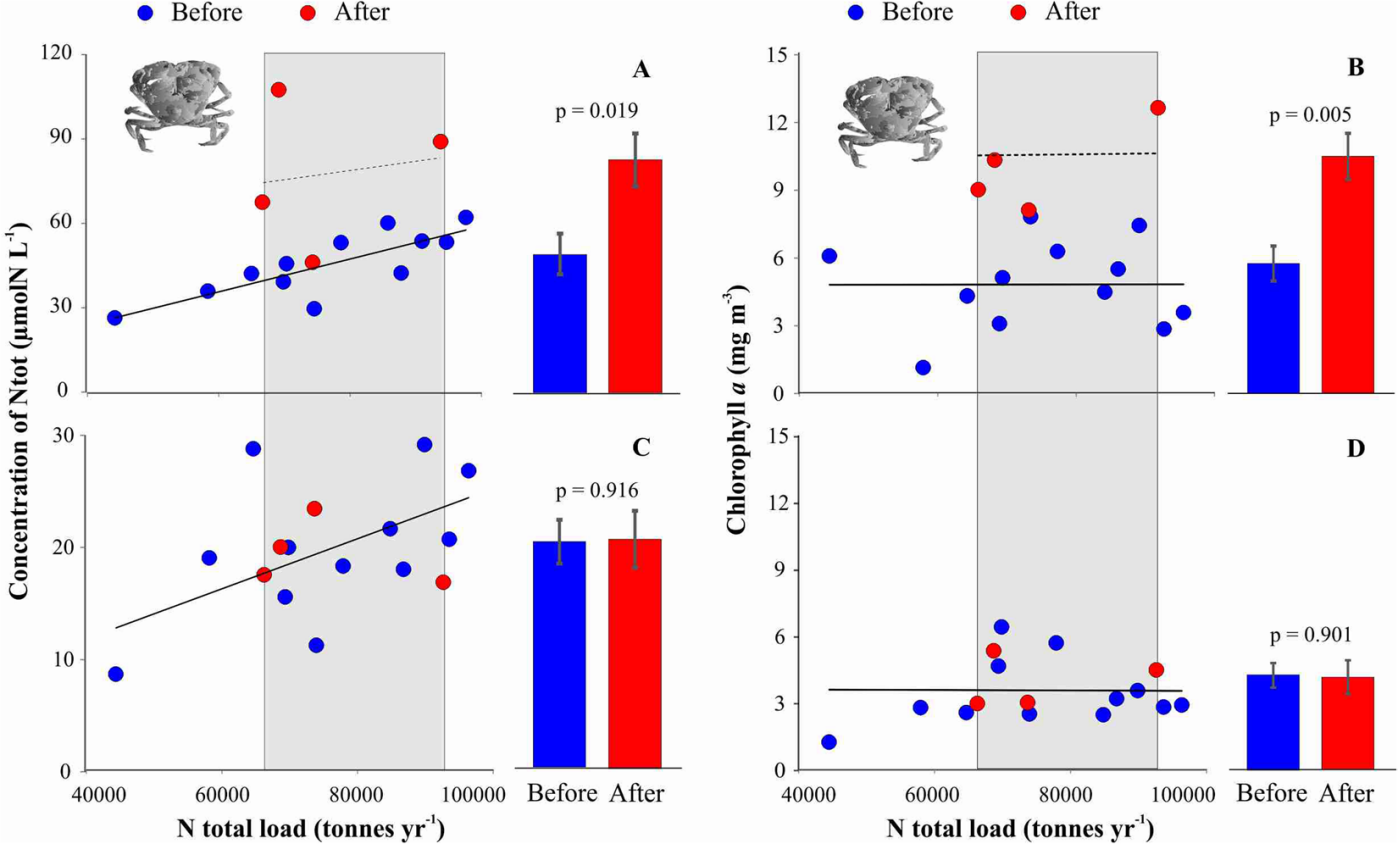
Relationship between annual nitrogen load, concentration of total nitrogen and chlorophyll *a* in seawater in pelagic habitat before and after crab invasion in crab-infested (**A**,**B**) and control areas (**C**,**D**). Fitted lines highlight the slope of the linear regressions. Grey area shows the nutrient load values after crab invasion and this range was used to compare changes in nutrient concentrations and the biota. Vertical bars denote 95% confidence intervals.

Similar to the benthic environment, there was a positive relationship between annual nitrogen loading and pelagic nitrogen, but not chlorophyll concentrations prior to the arrival of the crab (Fig. 2). Since the establishment of the mud crab, nutrient concentrations in seawater have doubled followed by a two-fold increase in the chlorophyll *a* concentration in the pelagic system (Fig. 2A,B). No such changes were observed in the control area lacking crabs (Fig. 2C,D).

**Figure 3:**
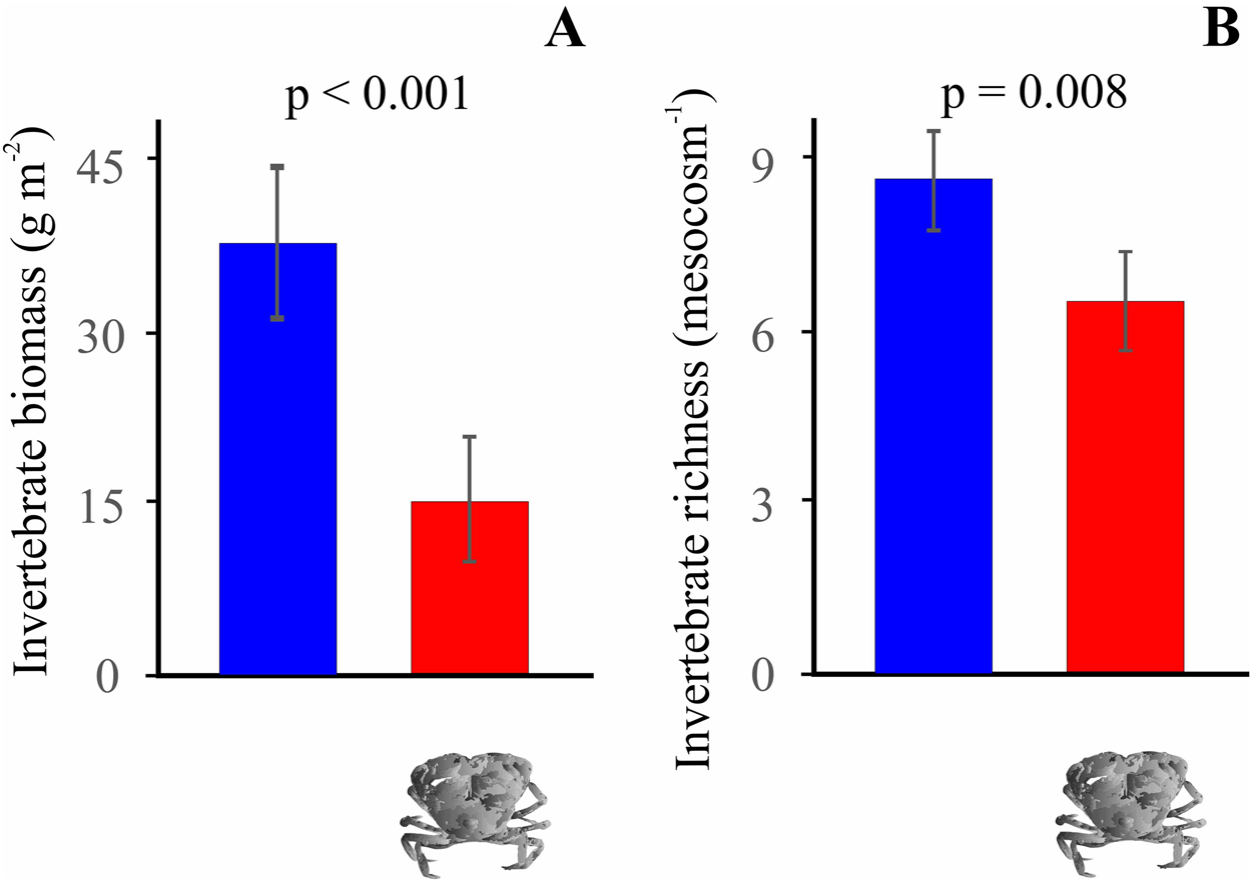
Results of the two-way ANOVA on the effect of mud crab on the total biomass (**A**) and richness (**B**) of benthic invertebrates in mesocosms. Vertical bars denote 95% confidence intervals. The other main effect (background nutrient level) and the interaction term (crab × nutrient level) were not significant at p < 0.05.

Controlled experiments with and without crabs supported the broad-scale observations of changes in benthic characteristics in areas with and without crabs. Under experimental conditions, mud crabs reduced the total biomass of benthic invertebrates regardless of nutrient enrichment scenario (Fig. 3A). Nutrient enrichment increased the total biomass of benthic invertebrates in the absence of crabs. No such enrichment effects were observed in the presence of crabs. These effects were mostly attributed to shifting biomasses of the dominant bivalves and gastropods (see Supplementary Appendix 3 online).

As in the field, there was no clear relationship between nutrient enrichment and invertebrate species richness in the experiment. Mud crabs reduced the richness of benthic invertebrates by ~22% irrespective of nutrient enrichment (Fig. 3B).

## Discussion

Here we showed how the introduction of a crab predator into an ecosystem previously lacking any similar ecological function, appeared to rapidly shift the balance between bottom-up and top-down control of production, causing a regime shift to an ecosystem state characterised by intensified symptoms of eutrophication. This transition is likely to be permanent as eradicating the crab is unrealistic.

Although the studied predator and prey are about the same size, our observations showed the invasive crab is able to effectively break the shells of the bivalves and gastropods within seconds only. Very high predator densities and per-capita predation rates^22^ likely triggered strong control over benthic communities, mostly of bivalves. Benthic deposit feeding clams together with suspension feeding mussels dominate in many temperate intertidal ecosystems including the Baltic Sea basin^26^. These bivalves feed extensively on deposited or suspended microalgae, and are responsible for the majority of energy fluxes within many benthic habitats, as well as fluxes between benthic and pelagic habitats^16,17^. Thus, it is plausible that a reduction of the bivalve populations by crabs, led to diminished nutrient capture and storage by benthic invertebrates, and increased pelagic nutrient availability ultimately boosting phytoplankton blooms.

Another indirect effect of the crab was a doubling of the biomass of *Dreissena polymorpha*, an invasive dreissenid bivalve, and the appearance of *Laonome* sp. nov^27^, an invasive polychaete. *Dreissena polymorpha* has much stronger shells than any of the native bivalves in the study area and predation on this invasive bivalve would be energetically costy for the crabs. Consequently, mud crabs presumably exert only a weak predation pressure on *D*. *polymorpha*. Moreover, the mud crabs indirectly increase phytoplankton biomass, providing food for the suspension feeding *D*. *polymorpha* and *Laonome* sp. and thereby create a good basis for the population growth of these invasive species. The range expansion of mud crabs and subsequent establishment of invasive suspension-feeding species provides an example of invasional “meltdown”, where the establishment of one invasive species in a new environment can facilitate the invasion of other non-native species^28,29^. A similar facilitation occurred in the Great Lakes during the 1980s when *D*. *polymorpha* was introduced into the region. Following their establishment they intensified benthic-pelagic coupling, improved water clarity and facilitated the invasion of exotic Eurasian watermilfoil *Myriophyllum spicatum*, but likely also many other nonindigenous and native plants^30^.

Consumer-mediated regime shifts in benthic marine ecosystems have occurred in coral reefs^3,31^ and kelp forests^32,33^. In these ecosystems, changes in herbivory directly affects primary producers and transforms the benthic ecosystem: tropical coral reefs change into turf- or seaweed-covered reefs^3,31^ and temperate kelp forests change into coralline barrens or turf landscapes^33,34^. Similarly, in freshwater ecosystems differences in trophic cascades explain differences in productivity among waterbodies with similar nutrient supplies. Here piscivores control the whole food web down to pelagic primary producers^35^. In contrast, the top-down cascade observed in this study is unique because it appears to drive a substantial shift in the benthic-pelagic coupling. This reveals that the profound ecological role of crab predation involves not only direct impacts on lower trophic levels but also propagates beyond the crab habitat, magnifying the effects.

The natural world is undergoing rapid changes due to intentional or unintentional alteration by humans. The accelerating redistribution of species through human-mediated introductions and climate change is driving the rise of communities with no past analogues, characterized by new species interactions and novel ecological functions^36,37^. As demonstrated in this study, these changes can disrupt the balance of food webs, causing fundamental transitions in ecosystems and thereby undermining important ecological services to humans (biodiversity, fisheries production, aesthetic qualities, etc.). This transition, however, does not yield stable solution as the studied ecosystem is currently being invaded by another aggressive benthic predator, the round goby *Neogobius melanostomus*^38^. Although, the habitats of these two novel species do not yet overlap, the invasion front of the round goby moves fast. In the invaded area the round goby can deplete local benthic invertebrate communities in a very short time^39^ and thereby further increase consumer control in food webs. Despite that mud crab prompted a severe reduction of the biomass of one invertebrate prey species only, the associated ecosystem effects were dramatic. These findings highlight the importance of species identity in food-web interactions but likely the current lack of understanding of interaction cascades triggered by non-indigenous species.

## Methods

### Study area

The Baltic Sea is a geologically young semi-enclosed sea and one of the largest brackish water basins in the world. Due to short evolutionary history and low salinity, it hosts very low numbers of benthic and pelagic species^40,41^.

This study took place in the Gulf of Riga, the north-eastern Baltic Sea within Estonian territorial waters. The study area has a salinity range of 2 to 7 psu and is highly eutrophicated. While the overall species richness is low, those species that inhabit the area often form very abundant populations. The coastal zone is extensive and benthic substrates consists primarily of a thin layer of slightly silted sand. Burrowing bivalves, amphipods, isopods and insect larvae have the highest biomass among invertebrates^20,41^.

### Field observations

To determine possible changes in benthic and pelagic communities as a consequence of the mud crab invasion, we compared benthic invertebrates in two comparable shallow water areas, with (Pärnu Bay) and without (Gulf of Riga) crabs, respectively (see Supplementary Appendix 1 online). The Gulf of Riga area has not yet been invaded (see result section) but is located less than 100 km from the nearest crab populations, and will likely be invaded within a few years. Every summer from 2000 to 2015, benthic invertebrates were sampled on soft bottom habitats by an Ekman-Lenz bottom grab (0.02 m^2^) in triplicate samples at 3 m depth.

Samples were sieved through 0.25 mm mesh screens in the field. The residues were stored at −20 °C and subsequent sorting, counting, and determination of invertebrate species were performed in the laboratory using a stereomicroscope. All organisms were identified to species level except for oligochaetes, juvenile gammarids, and insect larvae. The dry weights of species were obtained after drying the individuals at 60 °C for 2 weeks.

In addition, from June to August water samples for nutrients and phytoplankton were taken from the study areas fortnightly. Nutrient samples were frozen immediately until further laboratory analyses. Nutrients concentrations (phosphates (P-PO_4_) and nitrites + nitrates (N-NO_X_) were measured in laboratory with a continuous flow automated wet chemistry analyser Skalar SANplus using the methods EVS-EN ISO 11905-1:2003, EVS-EN ISO15681-2:2005, EVS-EN ISO 16264:2004 and EVS-EN ISO 13395:1999. Water samples for phytoplankton (1000 mL) were filtered through 0.45-μm Whatman GF/F filters, extracted in 96% ethanol overnight and the chlorophyll *a* concentrations were measured spectrophotometrically using Biochrom Libra S32.

Replicate samples of both benthic and planktonic biota were averaged to avoid pseudo-replication in statistical analyses and to provide a single value per year for each studied variable.

Data on the annual point and diffuse sources of total N and total P loads into the study area in 2000–2015 were obtained from the Estonian Ministry of the Environment and literature^42^. Discharges from point sources include municipal effluents, industrial effluents and pollution from fish farms. Diffuse sources of nutrients were defined as any anthropogenic sources of nutrients not accounted for as point sources, e.g., agriculture, forestry, storm water from built-up areas and atmospheric deposition to inland waters.

### Census of crab populations

Currently there is no monitoring programme for crab populations in Estonia. A census of crab densities along the coast of the Gulf of Riga was carried out at 8 sites in 2012, 2014 and 2015. In order to collect standardized samples, we used artificial collectors made of two hollow bricks (with a dimension of 28 × 8 × 5 cm and each brick having 20 different size holes) attached to each other by cable ties. The collectors were deployed at 2, 3 and 4 m at all 8 sites from June to August and provided habitat for natural recruitment of both juvenile and adult macroalgae and invertebrates. Collectors were retreived by divers placing a mesh bag gently around each collector before they were hauled to the surface for cleaning and collection of crabs. All crabs were counted and their length were measured using a vernier caliper.

### Field experiment

In order to test how adding a novel predator to the ecosystem modifies benthic communities and assess if these effects are modulated by background nutrient availability, a field enclosure experiment was undertaken in the northern Gulf of Riga site (see Supplementary Appendix 1 online) from June to August 2015 (62 days). In this season seawater temperature is above 5–10 °C, benthic communities are the most developed and crabs are active. Although nutrient loading is considered to be the main process that causes changes in the ecological structure and functioning of the experimental site^20^, this bottom up regulation is taking place only during summer and partly autumn months. This is why we carried out our experiment within the summer season to match the duration of productive season with the duration of experiment.

The mesocosms (10 L containers with a diameter of 24 cm) were filled with a 15 cm layer of sediment with associated benthic invertebrates, collected by the same sized corer from a shallow embayment adjacent to the experimental site, and allowed to settle for 6 hours. The experiment had two factors (factor levels in brackets): crabs (absent, present) and background nutrients (natural, increased), resulting in 4 treatment combinations, each replicated five times. One crab (average width of carapace was 1.3 cm corresponding to a typical length class of adult crabs in the study area) was added to each mesocom to achieve experimental densities (22 per m^2^) corresponding to densities of crabs in infested areas (see result section). In the Gulf of Riga crabs are relatively stationary with estimated home range at 0.004 to 0.04 m^2^ ^43^. This matches well with the spatial dimensions of mesocosms provided for crabs in the experiment (0.045 m^2^). Soft sediments contained silted sand. Nutrient addition was administered to mesocosms using commercial NPK fertilizer sticks by adding about 0.80 g N, 0.16 g P and 0.48 g K per mesocosm at the beginning of experiment. The added nutrient treatment simulated background nutrient conditions common in more eutrophicated embayments of the Gulf of Riga.

The containers were closed with mesh (0.5 cm mesh size) to avoid emigration of the crabs and at the same time assuring water exchange and immigration of benthic invertebrates from adjacent benthic communities. The experimental containers were distributed haphazardly on the seafloor at a depth of 1 m.

A procedural control assessing the effect of the mesh was also included. Comparison of screened and unscreened communities showed no significant effects of mesh on benthic communities (ANOVA, p > 0.05; see a section of data analyses below).

At the end of the experiment all mesocosms were gently retreived from the seafloor. Sediment and associated invertebrates were sieved through a 0.25 mm mesh sieve. The residues were stored at −20 °C before subsequent sorting, counting, and identification of invertebrates as described above.

All experiments were performed in accordance with relevant guidelines and regulations.

### Data analyses

In order to test how the coastal ecosystem responded to mud crabs, we performed linear regression analyses of the relationships between nutrient load, water nutrient concentration, phytoplankton biomass (chlorophyll *a*) as well as richness and biomass of benthic invertebrates separately before and after the establishment of mud crab. SIMPER analysis provided us the percent contribution of invertebrate species to the observed change in community composition in the crab infested area^44^.

For the mesocosm experiment, a two-way-ANOVA tested effects of crabs and background nutrient level on the biomass and richness of benthic invertebrates. Bartlett’s test was carried out prior to the analyses and the results confirmed the assumption of homoscedasticity^45^. Post-hoc Bonferroni tests were used to test which treatment levels were statistically different from each other. All univariate analyses were conducted using STATISTICA 7.0 software^46^. SIMPER analysis provided us the percent contribution of invertebrate species to the observed difference in community composition between mesocosms with and without mud crabs^44^.

### Data availability

The datasets that were generated and/or analysed during the current study are freely available from the corresponding author on a request.

## Electronic supplementary material

Supplementary information
