# Supplementary material for: Novel crab predator causes marine ecosystem regime shift
Source: Sci Rep. 2018 Apr 12;8:4956. doi: 10.1038/s41598-018-23282-w (PMC5897427; doi:10.1038/s41598-018-23282-w)
Supplement: Supplementary file 1 — Supplementary information [file 41598_2018_23282_MOESM1_ESM.pdf]

ONLINE MATERIAL FOR

Novel crab predator causes marine ecosystem regime shift

J. Kotta[*a], T. Wernberg[b], H. Jänes[a], I. Kotta[a], K. Nurkse[a], M. Pärnoja[a] and H. Orav-Kotta[a]

[a] Estonian Marine Institute, University of Tartu, Mäealuse 14, 12618 Tallinn, Estonia
[b] UWA Oceans Institute & School of Biological Sciences, University of Western Australia, Crawley WA6009, Australia

*corresponding author: jonne@sea.ee

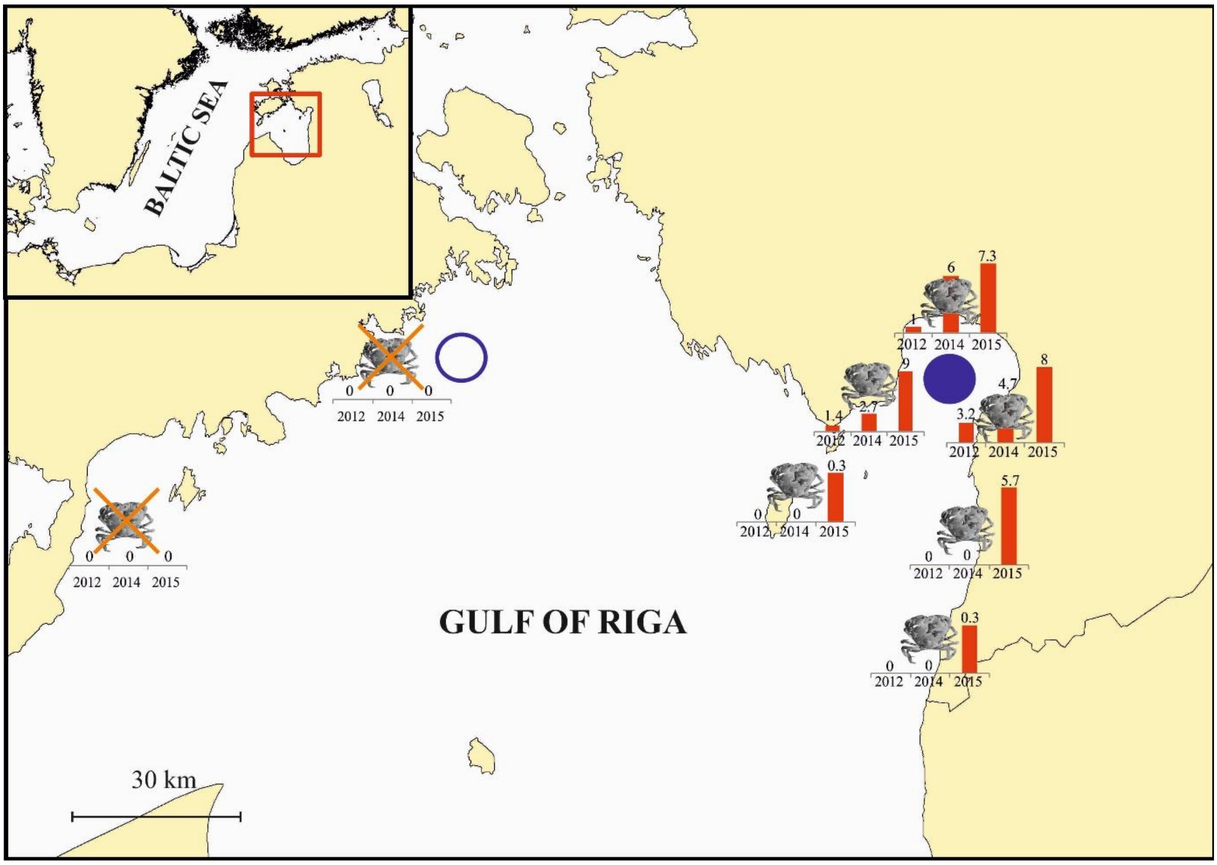

Appendix 1. Map of the sampling stations in the study area. Bar charts represent the locations of census of mud crab population with respective time-series of crab densities (ind per artificial collector). Filled circle (Pärnu Bay) indicates the sampling polygon of soft bottom and pelagic habitats of the impacted area with high mud crab density and empty circle (Gulf of Riga) the control polygon with no known record of the crab, respectively. The map of the Appendix 1 the map was generated using the software Corel Draw version X7 accessible at http://www.coreldraw.com/en/pages/patches/8100054.html.

Appendix 2. Mean biomasses (g dry weight m^{-2}) of benthic invertebrate taxa in the control and crab-infested area during pre- and post-invasion periods. SIMPER analysis shows the percent contribution of invertebrate species to the observed change in community composition in the crab infested area.

**Taxa**	**Control Before**	**Control After**	**Crab-infested Before**	**Crab-infested After**	**Contribution %**
Amphibalanus improvisus	0.000	0.000	2.168	1.520	2.97
Bathyporeia pilosa	0.000	0.000	0.002	0.000	<0.01
Bithynia tentaculata	0.087	0.587	0.000	0.000	0
Cerastoderma glaucum	16.003	13.514	0.983	0.185	2.58
Chironomidae	0.079	0.131	0.005	0.000	<0.01
Coleoptera	0.002	0.060	0.000	0.000	0
Corophium volutator	0.005	0.023	0.377	0.043	0.85
Cyanophthalma obscura	0.007	0.003	0.004	0.000	<0.01
Dreissena polymorpha	0.000	0.000	2.755	5.716	19.58
Ecrobia ventrosa	0.516	0.321	0.002	0.000	<0.01
Gammarus juv	0.051	0.010	0.003	0.000	<0.01
Gammarus oceanicus	0.025	0.228	0.000	0.000	0
Gammarus salinus	0.029	0.000	0.001	0.000	<0.01
Gammarus zaddachi	0.011	0.011	0.000	0.000	0
Gammarus tigrinus	0.210	0.419	0.001	0.000	<0.01
Halicryptus spinulosus	0.000	0.000	0.008	0.000	0.03
Hediste diversicolor	0.086	0.045	0.629	0.059	0.86
Idotea balthica	0.064	0.385	0.000	0.000	0
Idotea chelipes	0.146	0.117	0.000	0.000	0
Idotea granulosa	0.002	0.000	0.000	0.000	0
Jaera albifrons	0.004	0.017	0.000	0.000	0
Laonome sp. nov Kotta et al. 2015	0.000	0.000	0.000	0.440	0.07
Lepidoptera	0.000	0.006	0.000	0.000	0
Limecola balthica	5.761	5.337	47.826	16.141	56.36
Lymnaea stagnalis	0.000	1.208	0.000	0.000	0
Marenzelleria neglecta	0.000	0.000	0.239	0.116	0.22

Mya arenaria	0.001	0.000	4.860	0.295	16.16
Mytilus trossulus	0.766	2.083	0.000	0.000	0
Neomysis integer	0.004	0.000	0.004	0.001	<0.01
Odonata	0.132	0.043	0.000	0.000	0
Oligochaeta	0.000	0.005	0.314	0.052	0.18
Peringia ulvae	7.136	0.384	0.008	0.000	0.01
Physa fontinalis	0.000	0.016	0.000	0.000	0
Potamopyrgus antipodarum	0.057	0.000	0.001	0.000	<0.01
Radix balthica	0.395	0.690	0.000	0.000	0
Saduria entomon	0.000	0.000	0.443	0.095	0.06
Tenellia adspersa	0.000	0.007	0.000	0.000	0
Theodoxus fluviatilis	10.150	35.037	0.000	0.000	0
Trichoptera	0.004	0.135	0.000	0.000	0

Appendix 3. Mean biomasses (g dry weight m^{-2}) of benthic invertebrate taxa in the control and crab-infested mesocosms under natural and enriched nutrient conditions. SIMPER analysis shows the percent contribution of invertebrate species to the observed difference in community composition between mesocosms with and without mud crabs.

Taxa	Crab absent Natural nutrients	Crab absent Elevated nutrients	Crab present Natural nutrients	Crab present Elevated nutrients	Contribution %
Cerastoderma glaucum	5.579	7.079	2.728	1.160	17.87
Chironomidae	2.063	3.126	2.714	2.524	2.20
Coleoptera	0.022	0.027	0.000	0.000	0.10
Corophium volutator	0.000	0.000	0.000	0.002	<0.01
Cyanophthalma obscura	0.014	0.000	0.000	0.000	0.03
Gammarus juv	0.144	0.010	0.142	0.032	0.28
Gammarus oceanicus	0.003	0.000	0.000	0.004	0.01
Gammarus salinus	0.004	0.006	0.084	0.000	0.16
Gammarus tigrinus	0.184	0.262	0.259	0.585	0.90
Hediste diversicolor	0.501	0.000	0.251	0.199	1.05
Idotea baltica	0.000	0.035	0.018	0.000	0.07
Idotea chelipes	0.090	0.085	0.111	0.219	0.34
Limecola balthica	19.813	35.320	12.586	5.962	71.65
Marenzelleria neglecta	0.144	0.000	0.000	0.000	0.34
Mya arenaria	0.216	0.000	0.000	0.000	0.51
Peringia ulvae	0.174	0.052	0.090	0.067	0.27
Potamopyrgus antipodarum	0.119	0.000	0.002	0.000	0.28
Radix peregra	1.334	0.368	0.262	0.103	3.09
Theodoxus fluviatilis	0.142	0.341	0.000	0.040	0.84
